# Induction and recovery of copy number variation in banana through gamma irradiation and low‐coverage whole‐genome sequencing

**DOI:** 10.1111/pbi.12901

**Published:** 2018-03-24

**Authors:** Sneha Datta, Joanna Jankowicz‐Cieslak, Stephan Nielen, Ivan Ingelbrecht, Bradley J. Till

**Affiliations:** ^1^ Plant Breeding and Genetics Laboratory Joint FAO/IAEA Division of Nuclear Techniques in Food and Agriculture IAEA Laboratories Seibersdorf International Atomic Energy Agency Vienna International Centre Vienna Austria; ^2^ Present address: Department of Chromosome Biology University of Vienna A‐1030 Vienna Austria

**Keywords:** *Musa acuminata* Colla, LC WGS, gene dosage, mutation induction, deletion, indel

## Abstract

Traditional breeding methods are hindered in bananas due to the fact that major cultivars are sterile, parthenocarpic, triploid and thus clonally propagated. This has resulted in a narrow genetic base and limited resilience to biotic and abiotic stresses. Mutagenesis of *in vitro* propagated bananas is one method to introduce novel alleles and broaden genetic diversity. We previously established a method for the induction and recovery of single nucleotide mutations generated with the chemical mutagen EMS. However, officially released mutant banana varieties have been created using gamma rays, a mutagen that can produce large genomic insertions and deletions (indels). Such dosage mutations may be important for generating observable phenotypes in polyploids. In this study, we establish a low‐coverage whole‐genome sequencing approach in triploid bananas to recover large genomic indels caused by treatment with gamma irradiation. We first evaluated the commercially released mutant cultivar ‘Novaria’ and found that it harbours multiple predicted deletions, ranging from 0.3 to 3.8 million base pairs (Mbp). In total, predicted deletions span 189 coding regions. To evaluate the feasibility of generating and maintaining new mutations, we developed a pipeline for mutagenesis and screening for copy number variation in Cavendish bananas using the cultivar ‘Williams’. Putative mutations were recovered in 70% of lines treated with 20 Gy and 60% of the lines treated with 40 Gy. While deletion events predominate, insertions were identified in 20 Gy‐treated material. Based on these results, we believe this approach can be scaled up to support large breeding projects.

## Introduction

The continual improvement of crops is necessary to mitigate expanding pressures on food security such as increasing population, yield reduction due to climate change and changing food preferences (Ronald, 2011). Accessible genetic variation is a fundamental component of plant breeding as it provides a repository of alleles that can be exploited for developing novel traits for crop improvement and adaptation. Traditional breeding methods in seed propagated crops utilize natural nucleotide and chromosome structure differences within or between species to create new allelic combinations and alter heritable traits. Where sufficient variation is not available or accessible, mutations have been induced by treatment of plant cells with chemical mutagens or ionizing radiation (Jankowicz‐Cieslak *et al*., 2017). The use of mutagenesis to facilitate breeding has been termed ‘mutation breeding’, and to date, there are over 3275 officially registered mutant varieties with an estimated global economic impact in the billions of dollars (Ahloowalia *et al*., 2004; Jankowicz‐Cieslak *et al*., 2017; Kharkwal and Shu, 2009 2008).

Mutation‐based approaches can be especially useful in species with a narrow genetic base or those that are recalcitrant to traditional breeding methods. For example, major bottlenecks exist for obligate and facultative vegetatively (clonally) propagated species where meiosis is rare or nonexistent. Additionally, mutagens that cause dominant or hemizygous (dosage based) phenotypes can increase the efficiency of generating novel traits in polyploids as the expression of phenotypes arising from recessive mutations requires the combination of mutations from homeologous sequences (Krasileva *et al*., 2017). Gamma irradiation has been used widely as a mutagenizing agent for breeding programmes for many crops. The Joint FAO/IAEA Mutant Variety Database (MVD) lists 2519 officially released mutant crops produced by treatment with physical mutagens from which 1614 produced by gamma irradiation (FAO‐IAEA, 2017). Advances in genome sequencing technologies have allowed more precise evaluation of the effect of gamma irradiation on plant genomes (Li *et al*., 2016; Shirasawa *et al*., 2016). At higher dosages, the creation and repair of double‐strand breaks result in genomic insertions and deletions. For example, using exome capture sequencing in maize, Jia reported the recovery of a 6203‐bp deletion linked to opaque kernel phenotype (Jia *et al*., 2016). In poplar, treatment of pollen with gamma irradiation resulted in indels varying between whole chromosomes to small fragments (Henry *et al*., 2015). This work further showed that large genomic indels could be effectively recovered using low‐coverage whole‐genome sequencing (LC WGS), making mutation discovery more cost‐effective and data analyses more streamlined.

Large insertions and deletions lead to variation in copy number of many loci that can have profound effects on phenotypes of the organism. Copy number variations especially affect haploinsufficient genes for which a single functional copy of a gene is not sufficient for normal function (Birchler and Veitia, 2012; Casanova‐Sáez *et al*., 2014). Haploinsufficiency has been demonstrated to be induced by various stresses such as drugs, salt and high pH, and therefore may be important loci for improving crop resiliency to changing climates (Deutschbauer *et al*., 2005; Giaever *et al*., 2004; Lum *et al*., 2004). In addition, species that propagate asexually lack the mechanisms of meiosis and independent assortment to expunge deleterious alleles that arise in their genomes *via* spontaneous mutation. This phenomenon, known as Muller's ratchet, results in a high accumulation of heterozygous and potentially deleterious alleles as observed in species such as banana and potato (Jankowicz‐Cieslak *et al*., 2012; Muller, 1932; Potato Genome Sequencing *et al*., 2011). Single‐copy mutations, therefore, can potentially knock out the function of genes where only one functional copy is being maintained. Lack of meiosis also means that newly induced mutations will be more likely maintained in comparison with sexually propagated species. For example, in *Arabidopsis thaliana* (L.) Heynh. the majority of large deletions spanning millions of base pairs that are induced by treatment of pollen with gamma rays or carbon ion irradiation were not transmissible (Naito *et al*., 2005).

Bananas of the genus *Musa* L. are the fourth most important food crop and among the top ten food commodities for South‐East Asia, Africa and Latin America (FAO, 2018a). The majority of the edible cultivars of bananas are selected parthenocarpic diploid and triploid clones from the inter‐ and intraspecific hybridization of diploid seeded *Musa acuminata* Colla and *M. balbisiana* Colla (Perrier *et al*., 2011; Simmonds and Shepherd, 1955). However, at present only a few of the selected clones are having consumer‐preferred traits and dominate the global market. This genetic bottleneck has been perpetuated by tissue culture‐based mass clonal production, propagation and maintenance systems developed since the 1970s (Gowen, 1995). The most common export banana is the triploid *M. acuminata*. Banana plantations are comprised of nearly genetically identical cloned plants. The lack of genetic diversity poses the threat of mass devastation in the event of a pathogen attack, as occurred in the late 1890s and early 1900s with the outbreak of Panama disease, caused by soilborne fungus *Fusarium oxysporum f.sp. cubense* (Foc) Race 1. This led to a widespread epidemic and resulted in collapse of the cultivar ‘Gros Michel’ from the global market (Ploetz, 2005). Resistant Cavendish bananas maintained as specimens in the botanical gardens in the United Kingdom and in the United Fruit Company in Honduras were identified and used to replace ‘Gros Michel’ (Ploetz, 2006). However, Panama disease by Foc Tropical Race 4 (TR4) affecting these Cavendish cultivars emerged in late 1960s in Taiwan and has been spreading across borders. Reports of its spread outside of South‐East Asia to Jordon, Pakistan, Lebanon, Oman and Mozambique by 2013 have raised serious alarms as no commercial replacement for Cavendish bananas resistant to TR4 has been found yet (FAO, 2018b; García‐Bastidas *et al*., 2013; Molina *et al*., 2009). TR4 is potent, affects several local banana cultivars in addition to Cavendish types and can remain in soil for decades. Use of traditional fungicides to control TR4 has been largely unsuccessful. Its spread thus threatens not only the global export trade but also the local market and therefore can have a negative impact both on commercial economies and farmer's food security.

Inducing mutations in triploid *M. acuminata* provides an approach for generating genetic and phenotypic variation that can be clonally propagated. Previously, we established a system for inducing and maintaining SNP mutations induced by chemical mutagenesis. Treating shoot apical meristems of tissue cultured bananas with the chemical mutagen ethyl methanesulphonate (EMS) introduced a high density of GC‐AT transition mutations (Jankowicz‐Cieslak *et al*., 2012). We further showed that genotypic heterogeneity (also known as mosaicism or chimerism) caused by accumulation of different mutations in different cells of the plant propagule could be rapidly removed *via* isolation of shoot apical meristems and subsequent longitudinal bisection. We sought to establish a similar system for inducing and maintaining insertions and deletions. According to the MVD, only three mutant banana cultivars have been officially released. All three were produced by treatment with gamma irradiation. The cultivar ‘Al‐Beely’ showing 30% higher yield was released in 2007 in Sudan, while ‘Klue Hom Thong KU1’ with larger bundle was approved in 1985 for release in Thailand. ‘Novaria’, demonstrating early flowering, and improved fruit quality, was released in Malaysia in 1995 and is reported as the most commercially successful (Mak *et al*., 1996; Novák *et al*., 1990).

Screening for putative mutant candidates is an intensive step in any mutation breeding programme. We therefore aimed to develop an efficient pipeline for the generation and recovery of large copy number variations (CNV) in gamma‐irradiated Cavendish banana cultivars, employing tissue culture, low‐coverage whole‐genome sequencing (LC WGS) and chromosome dosage analysis. We chose a chromosomal dosage analysis that was previously successful in detecting aneuploidy, insertions and deletions in *Arabidopsis*, rice and poplar (Tan *et al*., 2015, 2016). To establish a pipeline for banana, we first adapted sequencing and dosage analysis for the previously released mutant banana cultivar ‘Novaria’. Large genomic deletions of up to 3.8 Mbps were recovered. We next developed a newly mutagenized banana population and tested two different irradiation dosages to establish that new genetic variation can be induced and maintained *in vitro*. This work suggests that a large‐scale mutagenesis pipeline can be created for routine production of mutant populations suitable for glasshouse and field evaluations.

## Results

### Establishing LC WGS dosage screening in banana mutant cultivar ‘Novaria’

**Figure 1:**
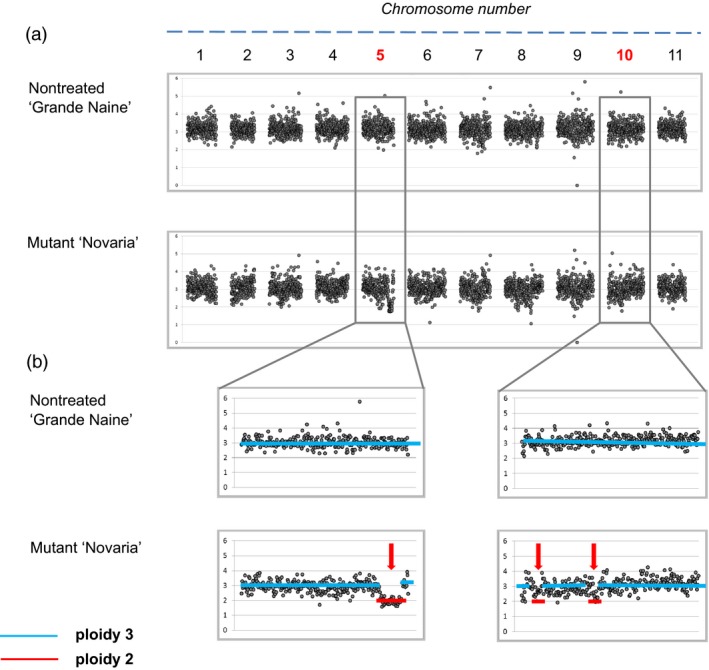
Relative sequence read coverage plots of nontreated ‘Grande Naine’ and mutant ‘Novaria’ for detection of gamma‐induced dosage variations. (a) Overview of relative sequence read coverage (RSRC) plots for all 11 chromosomes of all the samples together. (b) RSRC plots for selected chromosomes. RSRC values were set to 3.0 for the nontreated triploid control sample ‘Grande Naine’ #2 (GN2). Each bin of the other nontreated and mutant samples was compared to GN2. Lower RSRC values (<3.0) unique to mutant material indicate putative deletion of a chromosome fragment while higher values (>3.0) indicate putative additional copies due to insertional events in that region. Red arrows show selected regions of copy number variation due to deletion of a chromosomal fragment.

To establish recovery of large genomic indels induced by gamma irradiation, we used the officially released mutant cultivar ‘Novaria’. This triploid is derived from gamma irradiation of the cultivar ‘Grand Naine’ (AAA). *In vitro* plant material of both was acquired from the Bioversity International *Musa* Germplasm Transit Centre (ITC) and propagated to produce sufficient material for pilot studies. Low‐coverage whole‐genome sequencing was performed on four leaves from both cultivars using 2x300PE sequencing with an Illumina MiSeq. Between 4.8 and 7.4 million reads were produced for samples, generating between 2.20‐ and 3.47‐fold coverage (Table S1). Reads were aligned to the *Musa acuminata* reference sequence (D'Hont *et al*., 2012). A relative read coverage approach was used to discover variations in copy number in mutant samples whereby one nontreated ‘Grande Naine’ sample was chosen for all comparisons. Read coverage was normalized for a ploidy of three in each sample and then averaged over 100‐kb bins along the 11 chromosomes. Putative mutations were subsequently scored as deviations from copy number in nontreated ‘Grande Naine’ when variation was present in mutant ‘Novaria’ replicates (Figures 1, S1 and S2). Visual analysis of coverage graphs revealed putative deletions in ‘Novaria’ ranging between 0.3 and 3.8 million base pairs (Mbp). Analysis further suggested that all events were single copy with dosage changing from three to two. We next sought to evaluate mutation scoring *via* direct analysis of frequency coverage tables that are the output of the bioinformatics analysis. Ten putative deletions were called when using a filter whereby indels were called only if coverage deviated over three consecutive 100‐kb bins (Table 1). As expected with this approach, the smallest deletions recovered were 0.3 Mbp in size.

**Table 1:** Mutations identified in replicates of banana mutant cultivar ‘Novaria’

Chromosome	Start	End	Type	No. of 100‐kilobase bins	Approx. size (Mbp)^a^	No. of genes
1	26100001	26400000	Deletion	3	0.3	18
2	900001	1200000	Deletion	3	0.3	2
5	24400001	28200000	Deletion	38	3.8	129
8	12100001	12600000	Deletion	5	0.5	8
8	24100001	24400000	Deletion	3	0.3	3
9	14200001	14600000	Deletion	4	0.4	0
10	6000001	6300000	Deletion	3	0.3	3
10	12600001	12900000	Deletion	3	0.3	5
10	13000001	13400000	Deletion	4	0.4	9
11	12400001	12700000	Deletion	3	0.3	12

a Million base pairs.

### Validation of genomic deletions in ‘Novaria’

**Figure 2:**
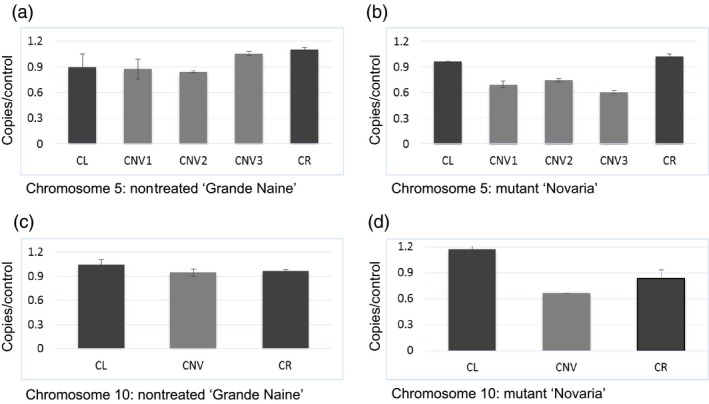
Quantitative real‐time PCR evaluation of DNA copy number. Panels (a) and (b) show data for chromosome 5 from nontreated ‘Grande Naine’ and mutant ‘Novaria’, respectively. CL and CR represent data from primers covering left and right boundaries outside of the predicted copy number variation region. Bars marked with CNV represent different regions internal to the predicted copy number variation region. Panels (c) and (d) show data for the 0.3‐Mbp deletion on chromosome 10. In both chromosomes, DNA quantity is reduced approximately 33% in the affected regions in ‘Novaria’.

**Figure 3:**
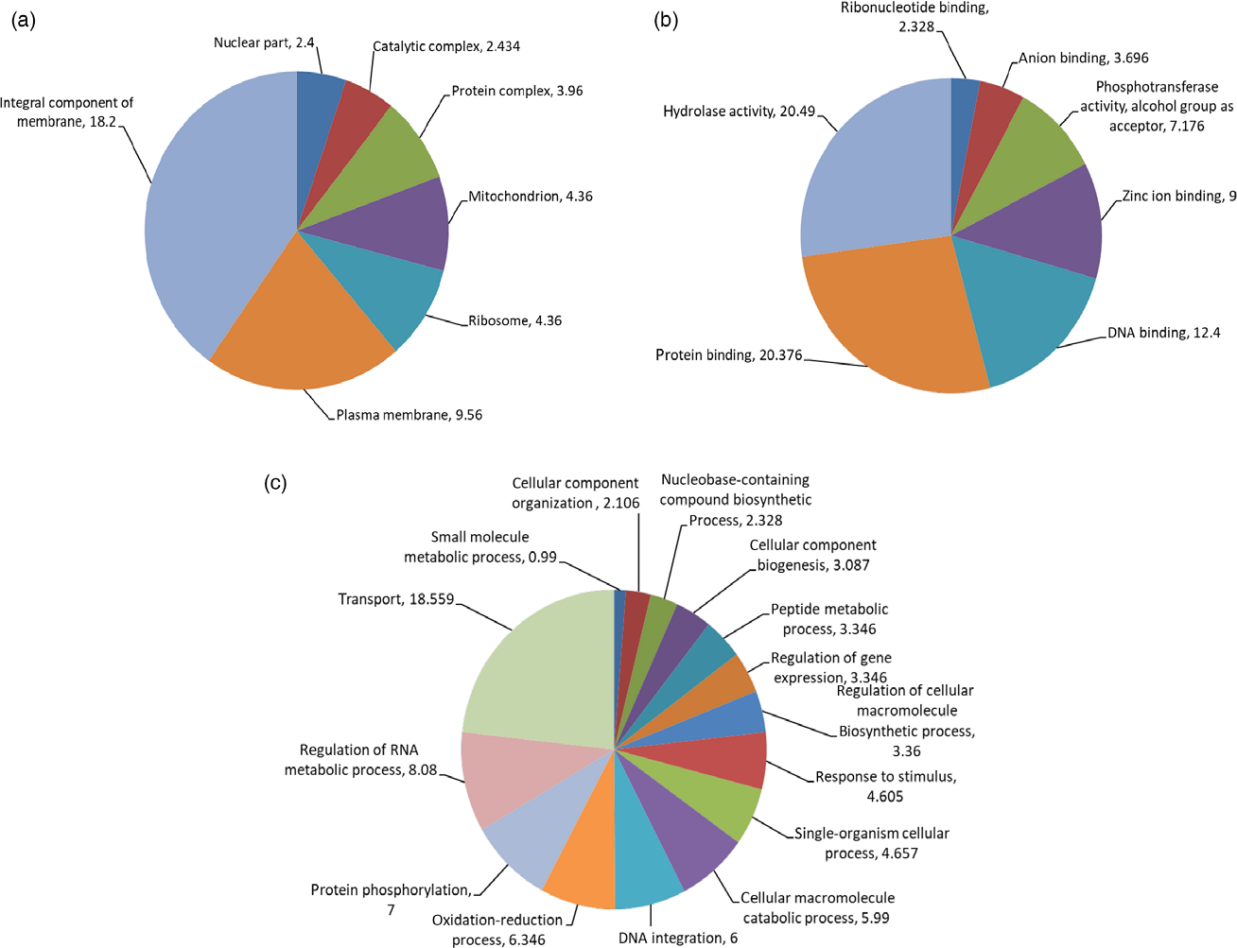
Gene Ontology annotations of genes in predicted CNV regions of mutant ‘Novaria’. Pie charts for cellular component (a) molecular function (b) and biological process (c) are shown.

To test the accuracy of mutation scoring by visual analysis and high stringency filtering approaches, we chose the largest and smallest deletions identified to evaluate copy number by quantitative PCR. Primers were designed in the boundary regions flanking the putative deletion events where copy number was predicted to be unaffected, and also within the region of the predicted deletion. In both chromosomes, an approximate 33% reduction in yield was observed in the affected region, suggesting the presence of single‐copy deletions as predicted by LC WGS assay (Figure 2; Table S2). We next investigated genes predicted to be present in the deleted regions. One hundred eighty‐nine genes fall within the affected areas (Tables 1 and S3). Gene Ontology (GO) analysis was performed to further evaluate affected regions (Figure 3; Table S4). This revealed genes involved in diverse processes such as gene expression, cellular biogenesis, protein phosphorylation, protein and DNA binding, and membrane components.

### Establishment of a pipeline for routine induction and screening of large indels in Cavendish bananas

**Figure 4:**
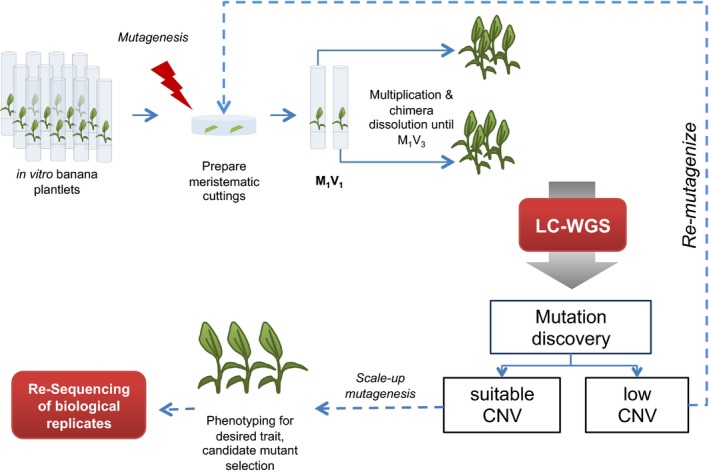
Schematic diagram of banana mutagenesis and recovery of mutations *via* LC WGS. Shoot tips isolated from banana plantlets propagated and maintained by shoot apical meristem culture were subjected to 20 or 40 Gy of gamma irradiation in bulk. Propagules were then recovered in groups of five and allowed to grow for approximately 4 weeks. Each plantlet at this stage is taken as the starting point of a mutant line to ensure unique mutation events in selected plantlets. The shoot tip of each plantlet is bisected and maintained in culture until growth of two leaves. The cycle is repeated for a minimum of three generations to remove genotypic heterogeneity resulting from mutagenesis of multicellular tissues. Leaves of plantlets from unique lines are then used for DNA extraction and subsequent whole‐genome sequencing. Chromosomal dosage analysis is carried out to detect indels. Once suitable mutagenesis conditions are established, the procedure can be scaled up for large‐scale plant phenotyping. Lines identified with desired phenotypes can be sequenced to develop dosage‐based molecular markers to enable sample tracking and validation in multilocation field trials.

Based on the results obtained with the previously released mutant ‘Novaria’, we developed a system for rapid mutation induction and screening of banana meristematic cultures using gamma irradiation that can be incorporated into a breeding programme (Figure 4). Briefly, triploid bananas of the cultivar ‘Williams’ were clonally propagated through bisection of shoot apical meristems in liquid culture. Two gamma dosages were applied towards the banana populations, 20 Gy and 40 Gy. Genetic mosaicism resulting from mutagenizing multicellular tissues was removed through repeated rounds of longitudinal bisection of the shoot apical meristem as previously described (Jankowicz‐Cieslak and Till, 2016, 2017).

**Figure 5:**
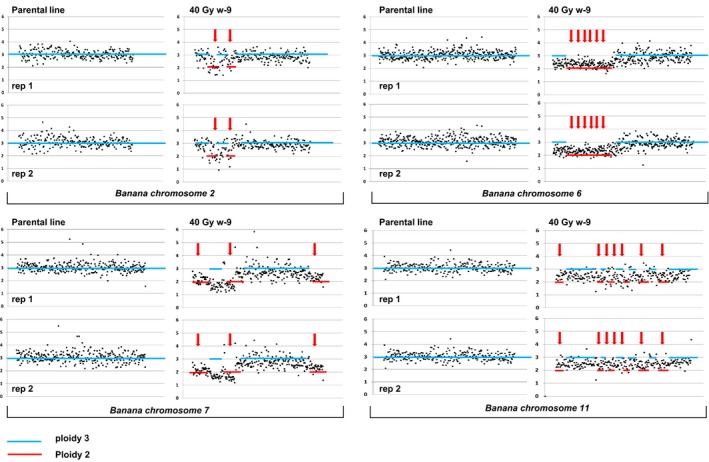
RSRC plots of chromosomes 2, 6, 7 and 11 of ‘Williams’ mutant line 40 Gy w‐9 and nonmutagenized parental material. Two mutant and three parental plants were evaluated. Plots were generated as in Figure 1. Mutations are marked with red arrows.

In order to quickly establish if a dosage treatment resulted in accumulation of novel mutations, we chose to evaluate a single plantlet per line. Leaves were isolated from 10 lines from each chosen treatment (20 Gy and 40 Gy). Whole‐genome sequencing and copy number variation detection analysis were applied using the graphical approach established for ‘Novaria’ (Table S5). Mutations in 7/10 lines treated with 20 Gy and 6/10 lines with 40 Gy could be quickly visually identified, suggesting both dosages are suitable for use in forward‐genetic screens (Figure S3). Dosage graphs also indicate the presence of putative single‐copy insertion events. In some lines, more than one mutation event could be visually detected. In total, we observe 10 events in 20‐Gy‐treated samples and nine events in 40‐Gy samples. Biological replicates were sequenced to further evaluate accumulation of mutations in gamma‐irradiated cultivar ‘Williams’. This controls for potential false positive signals resulting from endoreplication events. Mutant line 40 Gy w‐9 was propagated for four *in vitro* subcultures, and two clones were selected for LC WGS. All identified mutations were found in both replicates, indicating the gamma‐induced lesions are stably mitotically inherited (Figure 5). Analysis of frequency coverage output revealed 18 putative deletions accumulating in this line ranging from 0.3 to 6.8 Mbp. This covers a region containing 2762 predicted coding regions (Table 2).

**Table 2:** Mutations identified in replicates of ‘Williams’ banana mutant line 40 Gy w‐9

Chromosome	Start	End	Type	No. of 100‐kilobase bins	Approx. size (Mbp)^a^	No. of genes
2	3800001	4400000	Deletion	6	0.6	3
2	6600001	7000000	Deletion	4	0.4	9
6	3200001	3700000	Deletion	5	0.5	70
6	5600001	6300000	Deletion	7	0.7	142
6	6600001	7700000	Deletion	11	1.1	202
6	9300001	11700000	Deletion	24	2.4	479
6	12200001	12500000	Deletion	3	0.3	45
6	12800001	13400000	Deletion	6	0.6	103
7	100001	6900000	Deletion	68	6.8	1214
7	7100001	9300000	Deletion	22	2.2	128
7	26200001	28400000	Deletion	22	2.2	239
11	500001	800000	Deletion	3	0.3	54
11	7800001	8400000	Deletion	6	0.6	1
11	11300001	11600000	Deletion	3	0.3	8
11	13200001	13700000	Deletion	5	0.5	8
11	14300001	14600000	Deletion	3	0.3	5
11	16200001	16600000	Deletion	4	0.4	46
11	18000001	18300000	Deletion	3	0.3	6

a Million base pairs.

## Discussion

An important step in any programme employing induced mutations for functional genomics or trait development is the establishment of optimized parameters for the routine induction and recovery of mutations that can be applied to a large scale. This is especially important for clonally propagated crops such as banana where labour‐intensive *in vitro* culture is needed and phenotypic evaluation requires large greenhouse and field resources. Decreases in sequencing costs coupled with advances in next‐generation sequencing (NGS) offer a new avenue for improving the efficiency of banana trait development through induced mutations.

Large insertions and deletions provide several advantages when considering methods for improving polyploid banana. Simultaneously affecting the copy number of many genes can potentially lead to dosage‐dependent phenotypes and also reveal haploinsufficient loci. In the pilot screen, we recovered single‐copy genomic deletions in the commercially released ‘Novaria’ cultivar. More than 180 genes are predicted to be affected in 10 predicted deletion events. GO term analysis revealed genes involved in diverse processes such as gene expression and cellular biogenesis that may contribute to the phenotype of the cultivar. Further study is required to evaluate the effect of CNV on the expression of these genes and to estimate which targets may be causative for improved fruit quality and early maturity. While lack of meiosis and segregation makes establishing causality of mutations difficult, clonal propagation ensures mutations are genetically fixed and phenotypes are stable; two major advantages.

Another advantage to targeting large indels is that they can be efficiently discovered using LC WGS. In the pilot study of ‘Novaria’, we recovered deletions with approximately threefold coverage per sample. Such low‐coverage whole‐genome sequencing was previously shown to be an effective means of recovering CNV in poplar, rice and *Arabidopsis* and has been applied for evaluation of human clinical samples (Henry *et al*., 2015; Kader *et al*., 2016; Tan *et al*., 2016). In our pilot, we chose to evaluate four leaves from the ‘Novaria’ cultivar and four nontreated ‘Grande Naine’ samples. Biological replicates were included to control for presence of CNV arising from endoreplication events. Endoreplication is a phenomenon in which cell genomes can replicate without a subsequent cellular division and chromosome reduction (Gutierrez, 2016). This has been observed in both plants and animals, can be stimulated by environmental conditions, and is controlled by genetic and epigenetic mechanisms (Breuer *et al*., 2014; Hřibová *et al*., 2016; Lee *et al*., 2009). The result of endoreplication events are cells that can have altered copy number of loci, thus affecting the ability to discover CNV induced by gamma irradiation. It is possible to differentiate between endoreplication events and true mutations by sampling different tissues. Endoreplication events are expected to be cell linage specific and may be random in different cells with respect to chromosomal region. This means that CNVs resulting from endoreplication are not expected to occur in all tissues of a plant. Such variation is therefore not expected in multiple progeny resulting from clonal propagation. In contrast, after plants are propagated to remove chimeric sectors, all detected mutation events are expected in all tissues and propagates. In our pilot screen, we found no example of endoreplication as putative mutation events were identified in all clonally replicated material. Further, no evidence of endoreplication was found in biological replicates of a newly mutagenized cultivar ‘Williams’. Together, this suggests that interrogation of a single DNA sample (e.g. from a leaf) per mutant plant line is a suitable approach in banana.

To identify mutations in ‘Novaria’, we applied a dosage analysis method previously used for recovery of gamma‐induced indels in poplar, rice and *Arabidopsis* (Henry *et al*., 2015; Tan *et al*., 2015, 2016). We used a graphical approach and found that binning coverage into 100‐kb bins provided a rapid visual method for finding large indels in the banana genome. To evaluate the accuracy of this approach, we chose the largest (3.8 Mbp) and the smallest (0.3 Mbp) deletions and confirmed these to be single‐copy deletion events by qPCR. Single‐copy events are expected for a gamma‐induced deletion in mitotically propagated material. Validation of deletions by qPCR further shows the efficacy of using real‐time PCR for the development of dosage‐based molecular markers. In addition to being rapid, the analysis method is further advantageous in that direct comparisons are made between treated and untreated material and not to a published reference sequence. This is especially useful in cases where cultivars diverge significantly in sequence or structure from the published reference.

Owing to the fact that we did not observe endoreplication in ‘Novaria’, we chose to streamline our screening by sequencing only one sample per line in newly mutagenized material. This allowed seven mutants and one nontreated control to be sequenced on a single MiSeq run with a coverage of ~3.5×, similar to that used for screening ‘Novaria’. Sample number and throughput can be increased using a higher throughput Illumina sequencing platform. Based on our results, we estimate 30 banana plants can be sequenced per lane on a HiSeq 3000/4000 for a coverage of 5×. Sequencing biological replicates facilitates mutation calling. As sequencing costs drop, early identification of plants harbouring mutations will allow preselection of material for field evaluations based on affected genomic regions and/or specific coding sequences.

Induced mutations were relatively frequent in cultivar ‘Williams’ and were found in 70% of lines treated with 20 Gy and 60% of lines treated with 40 Gy. The observed rate of mutation accumulation in lines is slightly higher than the 50% recovery of mutations reported in gamma‐irradiated poplar (Henry *et al*., 2015). Interspecific comparisons are difficult as variations in the density of induced mutations are expected due to differential responses in mismatch repair and programmed cell death pathways (Fulcher and Sablowski, 2009; Yoshiyama *et al*., 2009). Higher mutation rates, however, are expected for polyploids, in part due to the buffering effect of additional nontreated ‘Grande Naine’ genomes (Birchler, 2012; Stadler, 1929). Further, lack of meiosis in vegetatively propagated bananas means that most lesions will be maintained.

Ten lines produced by treatment with 20 Gy and ten lines treated with 40 Gy were evaluated in this study. Visual detection resulted in 10 mutation events observed in 20 Gy‐treated material and nine events in 40 Gy‐treated material. While the sample size is too small to be significant, this trend is comparable to that reported for gamma‐irradiated rice where similar mutation rates were reported for seeds irradiated with 165 and 246 Gy (Li *et al*., 2016). However, an effect of dosage on mutation accumulation was reported for poplar and observed at higher dosages in rice (Henry *et al*., 2015; Li *et al*., 2016). Interestingly, putative insertion events were observed in two lines treated with 20 Gy, but none in material treated with 40 Gy. In addition, no insertion events were identified in ‘Novaria’. Further studies are required to determine whether irradiation dosage or genotype affects the accumulation of insertions or has an affect on the size of insertions or deletions.

When considering the large field resources needed to screen bananas, the ability to sequence a small number of individuals in order to fine‐tune mutagenesis should reduce costs and improve success rates. However, sequencing only one DNA sample per line likely results in an underrepresentation of the number of mutation events. For example, our analysis of two clones of a 40‐Gy‐irradiated ‘Williams’ revealed 18 putative deletion events covering approximately 20.5 Mbp of sequence. The inclusion of a biological replicate allowed the filtration of dosage fluctuations and enabled the scoring of more putative mutation events. This streamlines the analysis step as filtering rules can be applied to the output dosage table. In poplar, three consecutive bins with dosage variation were considered as true events (Henry *et al*., 2015). In banana, we tested this with the ‘Novaria’ data and found that deletions identified by graphical interface had at least three consecutive bins of similar dosage variation in the output dosage table for indel calling. We further set the filter value of ploidy in the dosage output tables to 3 (normal triploid state) and those in mutants to +/− 1, +/− 2 (corresponding to single‐ or double‐copy indel events) for identical mutation calling events in all samples. When using low‐coverage sequencing of biological replicates, however, false negative errors may be unavoidable. While it has been reported that larger indels predominate in gamma‐irradiated poplar and maize, an abundance of SNP and small indel variation has been observed in seed‐irradiated rice (Henry *et al*., 2015; Li *et al*., 2016; Yuan *et al*., 2014). Cataloguing all nucleotide variation requires higher coverage sequencing. Currently, this is cost‐prohibitive for evaluation of many samples.

In summary, we have established a pipeline for routine induction and recovery of genomic indels in polyploid banana that can be scaled up for large mutation breeding projects. Efficient pipelines for mutagenesis and recovery of deletion mutations will remain an important tool, especially in vegetatively propagated crops where dosage effects may be key for generating phenotypes such as Foc TR4 resistance. Mutagenized bananas are currently being evaluated for variations in Foc TR4 response (Till *et al*., 2016). Based on the results presented here, we propose a banana mutation breeding pipeline using gamma irradiation (Figure 4). We expect that further improvements and cost reductions in genome sequencing will make routine recovery of smaller indels and SNP mutations feasible in the near future, thus further expanding the resources for banana improvement.

## Experimental procedures

### Plant material

*In vitro* plantlets of banana (*Musa acuminata*) ecotype ‘Grande Naine’ (Cavendish AAA, ITC accession 0180) and ‘Novaria’ (Cavendish AAA, ITC accession 1329) were obtained from the *Musa* Germplasm Information System (MGIS, 2018) and ‘Williams’ (Cavendish AAA) from Du Roi Laboratory (Du Roi, 2018). Plants were maintained *in vitro* through mitotic propagation of shoot tips in liquid S27 media as previously described (Jankowicz‐Cieslak and Till, 2016; Jankowicz‐Cieslak *et al*., 2012).

### Mutagenesis

Shoot tips of *in vitro* ‘Williams’ plantlets were isolated in water. 10–15 shoot tips were bulked in a Petri dish and subjected to 20‐Gy or 40‐Gy gamma irradiation using a cobalt‐60 source (2.144 Gy/sec, Seibersdorf, Austria). Groups of five mutagenized shoot tips were placed in individual culture flasks and kept for recovery. Each shoot tip was used as the starting material for subsequent cultures and referred to as a line as previously described (Jankowicz‐Cieslak and Till, 2016; Jankowicz‐Cieslak *et al*., 2012). A minimum of three rounds of meristematic isolation and bisection were carried out to ensure removal of mutation‐induced genotypic heterogeneity (mosaicism) as was previously established (Jankowicz‐Cieslak *et al*., 2012).

### Library preparation and sequencing

DNA was extracted from leaf material using the DNeasy Plant Mini Kit (Qiagen, Cat Nr: 69106) according to the manufacturer's guidelines. Genomic DNA was assessed for quality and quantity using NanoDrop, agarose gel electrophoresis and Qubit fluorimeter as previously described (Duitama *et al*., 2017). Genomic libraries were prepared using the TruSeq^®^Nano DNA Library Prep Kit (version 15041110 Rev. D) following the recommended protocol for 550 bp fragment size. Dual indices were used, and the libraries were normalized to 4 nM prior to pooling. Seven libraries were pooled together for primary screening of ‘Novaria’ and mutated cultivar ‘Williams’, each pool containing a combination of mutant lines and nontreated controls. Nine libraries were pooled together for sequencing of 40 Gy‐treated ‘Williams’ line 9 (40 Gy w‐9) biological replicates. Sequencing was performed on an Illumina MiSeq using 2 × 300 Paired End version 3 chemistry.

### Data analysis

Raw data from the MiSeq were evaluated for the presence of large insertions and deletions as previously described (Henry *et al*., 2015). Briefly, the reads were first interleaved to form paired fastq files. A custom Python script (bwadoall) was then executed with default parameters (Comai, 2012). Bwadoall filters for quality, trims, aligns the reads against a given reference genome and converts the.sai files to give.sam files as output. The reference genome *Musa acuminata* v1 (Macuminata_304_v1) was used. Resulting BAM files are available at the Sequence Read Archive hosted by NCBI (SRA accession: SRP130725). Dosage plots were generated by running a custom Python script (BinbySam, 2013). The resulting dosage tables were then used to prepare graphs that were inspected for the presence of indels. Different binning sizes were evaluated, and the binning size of 100 kb was selected. To detect CNV in ‘Novaria’, data were compared to the nontreated ‘Grande Naine’ (GN), using two different approaches. First, the mean percentage values of all the individuals were used as a control. Secondly, a single GN sample was selected and all comparisons made to it. Similar dosage variation patterns were observed in both the methods (not shown), and so using a single reference sample was chosen for all subsequent analyses. The analysis of newly mutagenized cultivar ‘Williams’ material was performed by direct comparison to a selected nonirradiated ‘Williams’ control. Except for the parameter –p for ploidy, which was set at 3, default parameters were used in all other cases. Relative coverage values less than 3.0 indicate deletion of one or more copies of a chromosome fragment while higher values (>3.0) indicate potential insertional events. A dosage variation was considered to be true if at least three consecutive bins exhibited low or high relative coverage values. Read coverage and mapping statistics were generated from raw Bam files using QualiMap v.2.2.1 (Okonechnikov *et al*., 2016).

### qPCR evaluation of copy number

Selected CNV regions identified by sequencing were visualized using the integrative genomics viewer (IGV) in order to select regions falling within and outside the called predicted mutations (Robinson *et al*., 2011). Primers were designed using Primer3 with Tm between 65 and 72 °C (Rozen and Skaletsky, 2000). Primer sequences are listed in Table S6. qPCR was carried out on Qiagen Rotor Gene Q (Qiagen GmbH, Hilden, Germany) using the KAPA Library Quantification kits for NGS according to the manufacturer's guidelines and recommended temperature cycle settings except that kit‐supplied primers were substituted with designed primers. The average of the concentration of the qPCR product (copies per mL) in the unaffected regions of ‘Grand Naine’ and ‘Novaria’ was calculated. These averages were used for calculating the ratio of the concentration of the qPCR product for each sample at each region. These data were used for plotting graphs. Standard deviation was calculated for the ratios obtained from two to three replicates.

### Gene ontology analysis

Gene Ontology analysis was performed using Blast2GOBasic (Conesa and Götz, 2008). Sequences for genes in the regions of predicted CNV were acquired from the banana genome hub (BGH, 2018). Blast2GOdefault parameters were used to make multilevel pie charts.

## Conflict of interest

The authors declare that they have no conflict of interest.

## Supporting information


**Figure S1** Relative sequence read coverage (RSRC) plots showing mutation calls where dosage differences are detected in the mutant in at least three consecutive 100 kb bins.
**Figure S2** RSRC plots showing the presence of the 3.8 Mbp deletion identified in mutant ‘Novaria’ in 4 biological replicates.
**Figure S3** RSRC plots of newly mutagenized cultivar ‘Williams’. Data from 10 lines mutagenized at 20 and 40 Gy.

**Table S1** Raw read coverage statistics for cultivar ‘Novaria’ and cultivar ‘Grande Naine’.
**Table S2** Raw data from qPCR.
**Table S3** Annotated genes in the region of validated CNV regions in the ‘Novaria’ mutant cultivar.
**Table S4** Blast2GO results for regions of CNV in mutant ‘Novaria’ cultivar.
**Table S5** Raw read coverage statistics for cultivar ‘Williams’.
**Table S6** qPCR primer sequences.
