# Supplementary material for: Induction and recovery of copy number variation in banana through gamma irradiation and low‐coverage whole‐genome sequencing
Source: Plant Biotechnol J. 2018 Mar 24;16(9):1644–53. doi: 10.1111/pbi.12901 (PMC6097122; doi:10.1111/pbi.12901)
Supplement: Supplementary file 1 — Figure S1 Relative sequence read coverage (RSRC) plots showing mutation calls where dosage differences are detected in the mutant in at least three consecutive 100 kb bins. Figure S2 RSRC plots showing the presence of the 3.8 Mbp deletion identified in mutant ‘Novaria’ in 4 biological replicates. Figure S3 RSRC plots of newly mutagenized cultivar ‘Williams’. Data from 10 lines mutagenized at 20 and 40 Gy. [file PBI-16-1644-s002.docx]

Supplemental Figures for:

Induction and recovery of copy number variation in banana through gamma irradiation and low coverage whole genome sequencing

Sneha Datta^1†^, Joanna Jankowicz-Cieslak^1†^, Stephan Nielen^1^, Ivan Ingelbrecht^1^, and Bradley J. Till^1,2^*


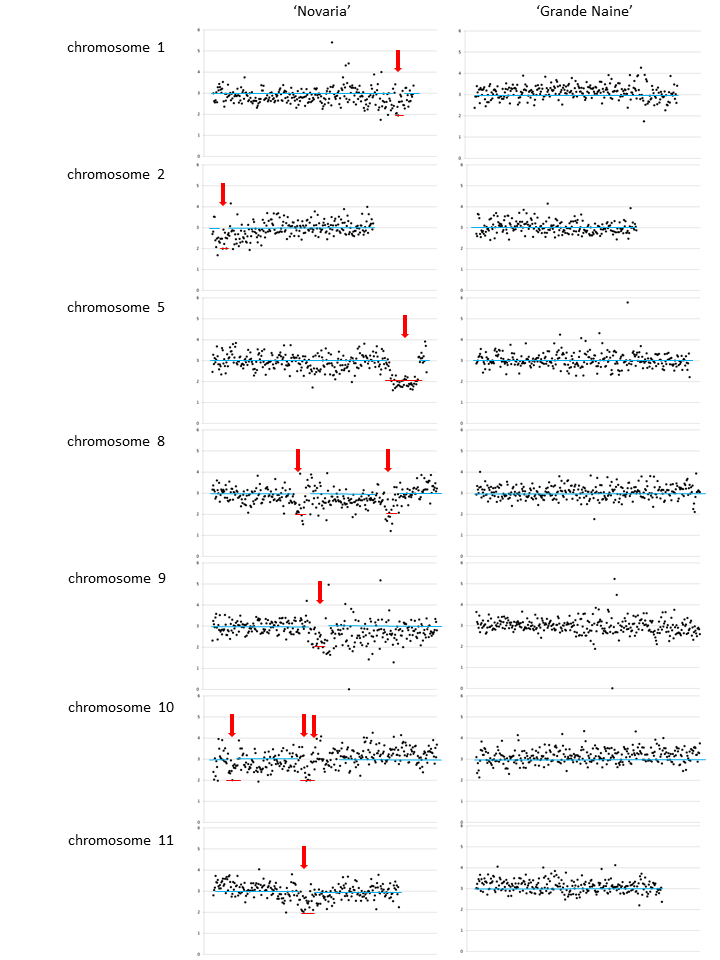


**Figure S1.** Relative sequence read coverage (RSRC) plots showing mutation calls where dosage differences are detected in the mutant in at least three consecutive 100 kb bins. Example calls are marked with red arrows


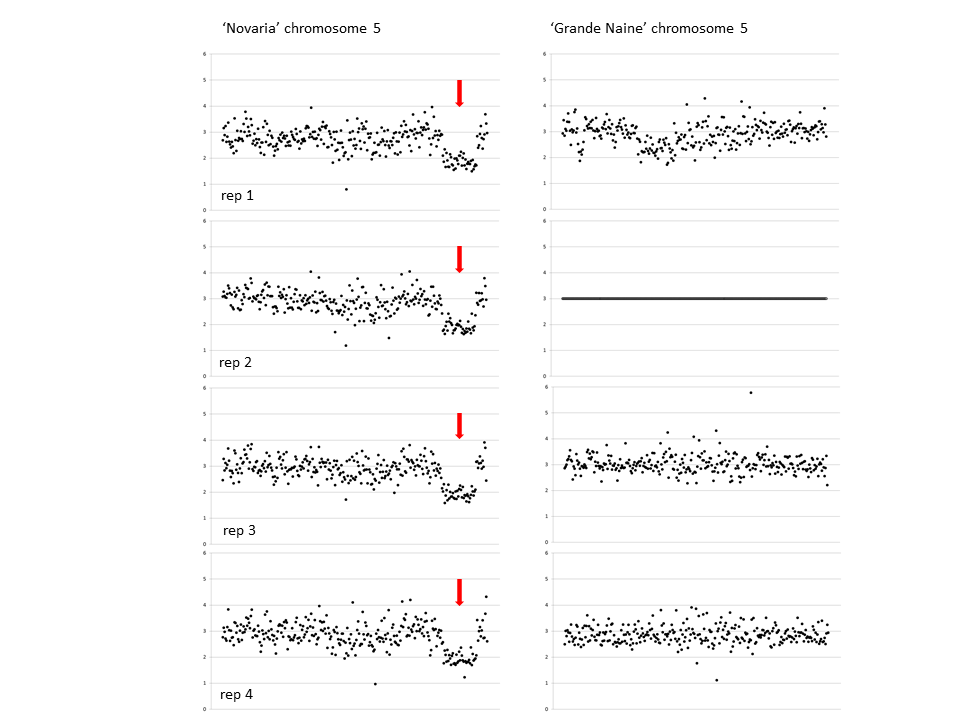


**Figure S2.** RSRC plots showing the presence of the 3.8 Mbp deletion identified in mutant ‘Novaria’ in 4 biological replicates (left). Plots of non-treated ‘Grande Naine’ are shown on right as a control. Grande Naine replicate 2 was selected as the sample for comparison. For this purpose, all reads for this sample are set to a copy number 3 and appear as a horizontal line.


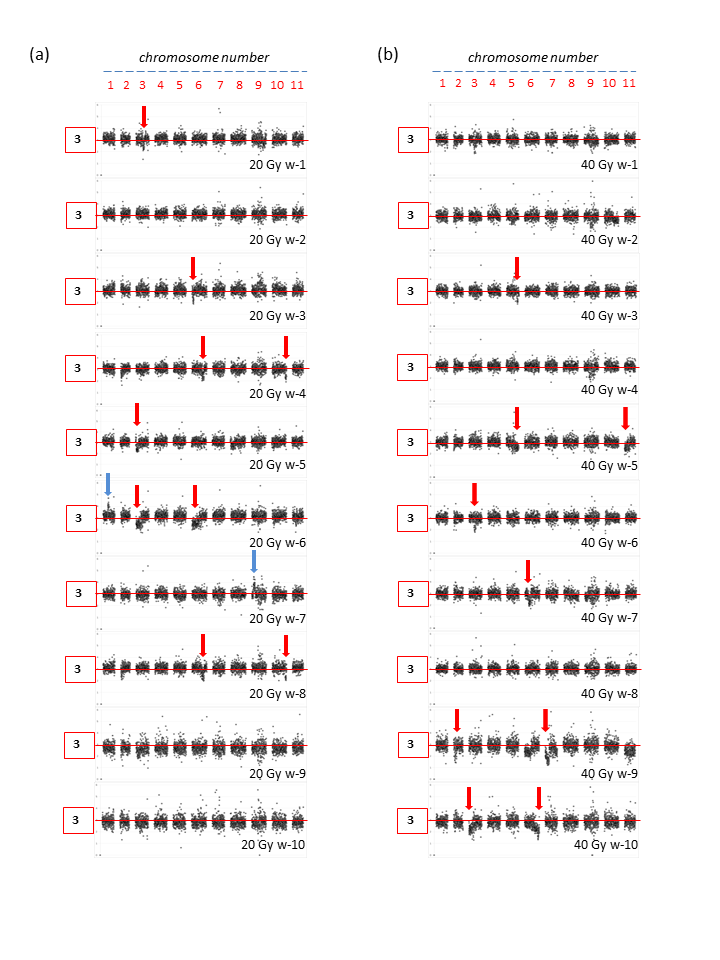


**Figure S3.** RSRC plots of newly mutagenized cultivar ‘Williams’. Data from 10 lines mutagenized at 20 Gy (a) and 40 Gy (b) are shown. Arrows mark selected regions of selected visually detectable putative CNV. Red and blue arrows represent deletion and insertions, respectively.
